# Responses to altered oxygen tension are distinct between human stem cells of high and low chondrogenic capacity

**DOI:** 10.1186/s13287-016-0419-8

**Published:** 2016-10-20

**Authors:** Devon E. Anderson, Brandon D. Markway, Derek Bond, Helen E. McCarthy, Brian Johnstone

**Affiliations:** 1 Oregon Health & Science University, 3181 SW Sam Jackson Park Road, HRC529C, Portland, OR 97239 USA; 2 Cardiff School of Biosciences, Sir Martin Evans Building, Cardiff, CF10 3AX UK; 3 Oregon Health & Science University, 3181 SW Sam Jackson Park Road, OP31, Portland, OR 97239 USA

**Keywords:** Physioxia, Hypoxia, Hypertrophy, Chondrogenesis, Articular cartilage progenitor cell, Mesenchymal stem cell, Pellet culture

## Abstract

**Background:**

Lowering oxygen from atmospheric level (hyperoxia) to the physiological level (physioxia) of articular cartilage promotes mesenchymal stem cell (MSC) chondrogenesis. However, the literature is equivocal regarding the benefits of physioxic culture on preventing hypertrophy of MSC-derived chondrocytes. Articular cartilage progenitors (ACPs) undergo chondrogenic differentiation with reduced hypertrophy marker expression in hyperoxia but have not been studied in physioxia. This study sought to delineate the effects of physioxic culture on both cell types undergoing chondrogenesis.

**Methods:**

MSCs were isolated from human bone marrow aspirates and ACP clones were isolated from healthy human cartilage. Cells were differentiated in pellet culture in physioxia (2 % oxygen) or hyperoxia (20 % oxygen) over 14 days. Chondrogenesis was characterized by biochemical assays and gene and protein expression analysis.

**Results:**

MSC preparations and ACP clones of high intrinsic chondrogenicity (termed high-GAG) produced abundant matrix in hyperoxia and physioxia. Poorly chondrogenic cells (low-GAG) demonstrated a significant fold-change matrix increase in physioxia. Both high-GAG and low-GAG groups of MSCs and ACPs significantly upregulated chondrogenic genes; however, only high-GAG groups had a concomitant decrease in hypertrophy-related genes. High-GAG MSCs upregulated many common hypoxia-responsive genes in physioxia while low-GAG cells downregulated most of these genes. In physioxia, high-GAG MSCs and ACPs produced comparable type II collagen but less type I collagen than those in hyperoxia. Type X collagen was detectable in some ACP pellets in hyperoxia but reduced or absent in physioxia. In contrast, type X collagen was detectable in all MSC preparations in hyperoxia and physioxia.

**Conclusions:**

MSC preparations and ACP clones had a wide range of chondrogenicity between donors. Physioxia significantly enhanced the chondrogenic potential of both ACPs and MSCs compared with hyperoxia, but the magnitude of response was inversely related to intrinsic chondrogenic potential. Discrepancies in the literature regarding MSC hypertrophy in physioxia can be explained by the use of low numbers of preparations of variable chondrogenicity. Physioxic differentiation of MSC preparations of high chondrogenicity significantly decreased hypertrophy-related genes but still produced type X collagen protein. Highly chondrogenic ACP clones had significantly lower hypertrophic gene levels, and there was little to no type X collagen protein in physioxia, emphasizing the potential advantage of these cells.

**Electronic supplementary material:**

The online version of this article (doi:10.1186/s13287-016-0419-8) contains supplementary material, which is available to authorized users.

## Background

Articular cartilage has limited potential to repair or regenerate following acute injury and chronic degeneration due to lack of a blood supply, low cell density, and highly organized extracellular matrix. Tissue engineering strategies to repair articular cartilage remain limited by our inability to differentiate cells in vitro toward the stable articular cartilage tissue phenotype, which is characterized by high type II collagen and aggrecan and low type I and type X collagen in the extracellular matrix. Human bone marrow-derived mesenchymal stem cells (MSCs) are an attractive candidate as an autologous cell source for tissue engineering application because they are easily harvested through bone marrow aspiration from adults; however, MSCs invariably progress toward the hypertrophic phenotype during in-vitro chondrogenesis [1, 2].

The physiologic oxygen tension (physioxia) within tissues in the human body is well below the atmospheric level (hyperoxia), with the highest level in the alveoli (110 mmHg) and arterial blood (100 mmHg) [3]. Articular chondrocytes live in a physiologic environment of 1–5 % (8–40 mmHg) oxygen, and bone marrow resides in approximately 7 % oxygen (50 mmHg) [3–5]. It is well known that oxygen deprivation in tissues stabilizes expression of hypoxia-inducible factors (HIFs), which directly modulate in-vitro chondrogenesis through transactivation of regulatory transcription factors, including sex determining region Y-box 9 (SOX9)—the master regulatory transcription factor for chondrogenesis [6, 7]. Oxygen-mediated mechanisms drive anabolism of the extracellular matrix molecules toward the articular cartilage phenotype, but the role of physioxia remains equivocal with regard to MSC terminal differentiation toward the hypertrophic phenotype.

We have found that genes of the hypertrophic phenotype, *COL10A1* and *MMP13*, and of the fibrocartilaginous phenotype, *COL1A1*, are significantly lower in both healthy and osteoarthritic adult human chondrocytes during redifferentiation in low-oxygen culture, which further increases proteoglycan production and promotes expression of cartilage matrix genes, including *COL2A1* and *ACAN* [8]. The effect of lowered oxygen tension on markers of hypertrophy during chondrogenic differentiation of bone marrow-derived MSCs is less clear, with results ranging from downregulation [9–13] to no change [14–16] to upregulation [17, 18] of *COL10A1* and/or *MMP13*. In our own previous work, we found that while proteoglycan production and *COL2A1* and *ACAN* expression are promoted in MSCs, *COL10A1* expression is enhanced rather than suppressed in low-oxygen culture [17]. These studies, however, were conducted using MSCs that had been expanded without FGF-2 supplementation, which is known to improve subsequent chondrogenesis [19–21], and the pellets exhibited poor chondrogenesis regardless of oxygen tension. In our more recent studies, using highly chondrogenic preparations, MSCs cultured at low oxygen downregulated hypertrophic genes [12].

Articular cartilage progenitor (ACPs) cells are a cell population that exists in the upper layer of mature articular cartilage. They have generated significant interest with regard to their role in tissue development [22–24], in-situ response to injury [25–29], and tissue engineering [30–33]. Increasing evidence suggests that ACPs generate stable articular chondrocytes of native tissue through appositional growth of clonal populations [24]. In vitro, clonal ACPs undergo chondrogenic differentiation with reduced potential for terminal differentiation toward the hypertrophic phenotype, in contrast to MSCs [31]. Further, chondrogenic potential is maintained with extended population doublings and reduced telomere shortening in subclonal populations [34]. Although ACPs reside in a low-oxygen environment in vivo, where oxygen tension likely influences both differentiation and subsequent tissue homeostasis, the data concerning their differentiation were all generated in a hyperoxic environment of 20 % oxygen in vitro.

While adult stem cells, including bone marrow-derived MSCs and tissue-derived ACPs, are promising cell candidates for autologous tissue regeneration, there exists substantial heterogeneity across populations of cells from adult human donors [10, 35–38]. Generating clonal populations of MSCs is technically very challenging. Among the few successful examples, clonal MSC populations derived from individual human donors demonstrate intraclonal heterogeneity with respect to proliferative efficiency, differentiation capacity, and phenotype [39, 40]. In contrast to MSCs, ACPs are clonable, but intradonor variation has only been defined at the level of colony-forming efficiency [30], and intraclonal variation remains undefined. Without standardized cell isolation and differentiation protocols in articular cartilage tissue engineering, generalized comparisons across and within cell populations from adult human donors, especially when pooled from multiple donors, may hinder our ability to identify subsets of cells with which to effectively generate autologous tissue applicable to adult patients.

The objective of the current study was to define the influence of oxygen tension on chondrogenic differentiation, specifically gene and protein expression, of adult MSCs and ACPs. In particular, we focused on intradonor variability of MSCs and both intradonor and intraclonal variability of ACPs. We reasoned that not all adult human stem cells are equivalent, and there exists a range of chondrogenic potential for cells derived from different biologic donors as well as for cells derived from clonal populations from a single donor. This may have consequences for understanding the response of stem cell populations to biological stimuli; for example, the discrepancies in reports on the effects of oxygen on MSC hypertrophy. Thus, we investigated whether stem cell responses to changes in oxygen level during differentiation depends on intrinsic chondrogenic capacity.

## Methods

### Cell harvest and isolation

Human MSCs were isolated from iliac crest bone marrow aspirates and expanded in monolayer culture as described previously [1, 12]. Briefly, bone marrow aspirates were obtained, with approval from the Institutional Review Board at Oregon Health & Science University, Portland, OR, USA (IRB00000605), from 14 consenting donors (nine females, five males, age 46–74) and fractionated on a Percoll density gradient. Cells were plated at 160,000 cells/cm^2^ in low-glucose Dulbecco’s modified Eagle medium (DMEM; Grand Island, NY, USA) with 10 % (v/v) fetal bovine serum (FBS) and 1 % (v/v) penicillin–streptomycin (P/S). Adherent cells were cultured at 37 °C, 5 % CO_2_ (atmospheric oxygen) with medium changes every 3–4 days until confluent, at which point expansion medium was supplemented with 10 ng/ml basic fibroblast growth factor (FGF-2; PeproTech, Rocky Hill, NJ, USA).

Normal articular cartilage from the femoral condyle of four healthy human donors (all male, age 26–33) was obtained with relevant ethical approval and informed written consent from the NHS Blood and Tissue bank, Liverpool, UK (NRES number: 09/WSE04/35). Chondrocytes from the full thickness tissue were isolated through a sequential pronase (70 U/ml for 20 min at 37 °C) and type I collagenase (300 IU/ml for 4 h at 37 °C) digestion. Immediately following digestion, colony-forming cells were isolated from the chondrocyte population through differential adhesion to fibronectin [30]. A total of 18 clones from four biologic donors were evaluated. Cells were expanded in DMEM/F12 (1:1) medium containing 1 mg/ml l-glucose, 10 mM HEPES, 10 % (v/v) FBS, 1 % (v/v) P/S, 0.1 mM ascorbic acid 2-phosphate, 1 ng/ml transforming growth factor β1 (TGFβ1; PeproTech), and 5 ng/ml FGF-2 (PeproTech).

### Pellet culture

Chondrogenic differentiation was induced in serum-free high-glucose DMEM containing 10 ng/ml TGF-β1 (PeproTech), 10^–7^ M dexamethasone (Sigma), 37.5 μg/ml ascorbic acid 2-phosphate (Wako), 1 mM sodium pyruvate, 40 μg/ml l-proline, 1 % (v/v) ITS+ Universal Culture Supplement Premix (BD Biosciences, San Jose, CA, USA), and 1 % (v/v) P/S [1]. Pellet cultures were formed by centrifuging 5 × 10^4^ cells (MSCs) or 1 × 10^5^ cells (ACPs) at 500 × *g* for 5 min in 240 μl of medium in Nunc polypropylene V-bottom 96-well plates (Thermo Fisher Scientific, Waltham, MA, USA). Cultures were maintained in a low-oxygen incubator (Thermo) set at 2 % oxygen and 5 % CO_2_ (physioxia), or in a standard tissue culture incubator at 20 % oxygen and 5 % CO_2_ (hyperoxia). By convention, we refer to the atmospheric, thus hyperoxic, condition as 20 % oxygen. Medium was changed every 2–3 days, with that of the physioxic cultures being done at 2 % oxygen in a low-oxygen chamber (BioSpherix, Lacona, NY, USA) using medium pregassed to 2 % oxygen, ensuring cells consistently saw that level of oxygen for the entire experimental period.

### Biochemical analysis

Triplicate pellets from each condition were rinsed with phosphate-buffered saline (PBS) and digested overnight at 60 °C in 4 U/ml papain (Sigma-Aldrich, St Louis, MO, USA) in PBS containing 6 mM Na_2_-ethylenediaminetetraacetic acid and 6 mM l-cysteine (papain buffer, pH 6.0).

Total DNA and sulfated glycosaminoglycan (GAG) content were quantified using Hoechst and 1,9-dimethymethylene blue (DMMB) assays, respectively [8]. The DNA content of pellet digests was quantified using calf thymus DNA diluted in papain buffer as a standard in serial dilution. Diluted samples, standards, and blanks were added to Hoechst dye (2 μg/ml), and fluorescence emission was measured with a multiwell plate reader (excitation 355 nm, emission 455 nm).

Supernatant was collected at each medium change to quantify the total amount of GAG produced and lost into the medium. GAG content of pellet digests and supernatant was quantified using shark chondroitin sulfate (Sigma-Aldrich) diluted in either DMEM or papain buffer as a standard in serial dilution. DMMB dye (18 μg/ml in 0.5 % ethanol, 0.2 % formic acid, 30 mM sodium formate; pH 3.0) was added to samples, standards, and blanks, and absorbance was measured (575 nm).

Each cell type, MSCs and ACPs, was divided into two groups based on GAG production in hyperoxia relative to human chondrocytes cultured in standard chondrogenic pellet culture conditions (*n* = 4 each of 5 × 10^4^ and 1 × 10^5^). Preparations or clones within two standard deviations of GAG production for the human chondrocytes were considered high-GAG, and those below two standard deviations were considered low-GAG.

### Gene expression analysis

RNA was isolated from six pellets of each replicate in each condition. Briefly, pellets were pooled, snap frozen directly in liquid nitrogen, crushed with a mini pestle, and lysed with Buffer RLT (QIAGEN, Germantown, MD, USA) containing 40 mM dithiothreitol (DTT). RNA isolation was performed with the RNeasy Mini Kit (QIAGEN) according to the manufacturer’s instructions. Total RNA was quantified on a NanoDrop Spectrophotometer (NanoDrop, Wilmington, DE, USA) and 250–500 ng was reverse-transcribed using qScript cDNA SuperMix (Quanta BioSciences, Gaithersburg, MD, USA). Quantitative polymerase chain reaction (qPCR) was performed with a 1:10 dilution of cDNA using a StepOnePlus thermal cycler (Life Technologies, Grand Island, NY, USA) with TaqMan Fast Advanced Master Mix and the following TaqMan assay primers (Life Technologies): *COL2A1* (Hs00264051_m1), *COL9A1* (Hs00932129_m1), *COL11A2* (Hs00365416_m1), *COL6A1* (Hs00242448_m1), *COL1A1* (Hs00164004_m1), *COL10A1* (Hs00166657_m1), *SOX9* (Hs01001343_g1), *L-SOX5* (Hs00374709_m1), *SOX6* (Hs00264525_m1), *ACAN* (Hs00153936_m1), *PRG4* (Hs00195140_m1), *LOX* (Hs00942480_m1), and *MMP13* (Hs00233992_m1). Cycling parameters were 50 °C for 2 min, 95 °C for 20 seconds, and then 95 °C for 1 second and 60 °C for 20 seconds for a total of 40 cycles. Results were analyzed using the 2^–ΔCt^ method relative to *18S* (Hs99999907_m1) housekeeping gene—the most stably expressed of four evaluated housekeeping genes across all experimental replicates and between groups. Gene expression in physioxia was normalized to the paired gene in hyperoxia to calculate the relative fold-change expression.

To evaluate a panel of oxygen tension-mediated genes, RNA was pooled at equal quantities from each group, and 5 ng of cDNA was loaded into each well of a human TaqMan gene array (catalog number #4414090; Life Technologies). Results were analyzed using the 2^–ΔCt^ method relative to *18S* housekeeping gene, consistent with single gene analysis. A cycle threshold of 36 was defined for minimum expression levels across the panel, and undetectable genes above the threshold were not evaluated. Gene expression in physioxia was normalized to the paired gene in hyperoxia to calculate the relative fold-change expression. Heatmaps were generated in RStudio (Boston, MA, USA): one without scaling to reflect the fold-change expression; and one with a *z*-score cluster analysis. Gene relationships were defined using the STRING (Search Tool for the Retrieval of Interacting Genes/Proteins) database v10, and genes were grouped according to common biological processes of gene ontology.

### Histology and immunohistochemistry

Pellets were fixed in 10 % neutral buffered formalin, embedded in paraffin, and sectioned onto slides. Proteoglycan content was visualized by histochemical staining with toluidine blue (0.04 % toluidine blue, 0.2 M acetate buffer, pH 4.00) after deparaffinization. Pellet size was determined by measuring the widest diameter with digital analysis of toluidine blue-stained sections taken from the middle of the pellet (ZEN blue; Ziess, Oberkochen, Germany).

For immunohistochemistry, sections were deparaffinized and pretreated for antigen retrieval. For type X collagen, sections were treated with 1 mg/ml protease (Roche) in PBS for 30 min at room temperature. For type I and type II collagen, protease treatment was followed by 1 mg/ml hyaluronidase in PBS for 30 min at 37 °C. Sections were blocked with a blocking buffer composed of 4 % normal goat serum (NGS), 1 % bovine serum albumin (BSA), and 0.3 % Triton X-100 in 1× PBS, and were subsequently probed with primary antibodies to type X collagen (mouse polyclonal antibody, 1:300; kind gift from GJ Gibson, Henry Ford Hospital, Detroit, MI, USA), type I collagen (rabbit polyclonal antibody, 1:200; kind gift from A Hollander, University of Bristol, Bristol, UK), and type II collagen (II-II6B3 mouse monoclonal antibody, 1:200; Developmental Studies Hybridoma Bank, University of Iowa, Iowa City, IA, USA) overnight at 4 °C. Secondary antibodies, including Oregon Green-conjugated anti-rabbit (1:250) and Alexa Fluor 596-conjugated anti-mouse (1:250) (Life Technologies) in blocking buffer, were incubated on the slides for 45 min at room temperature. Slides were mounted with ProLong Gold antifade reagent containing 4′,6-diamidino-2-phenylindole (DAPI; Life Technologies) and imaged at 20× using a Leica DM4000b upright fluorescent microscope (Leica Microsystems, Buffalo Grove, IL, USA).

### Western blotting

To confirm the presence of protein visualized in immunohistochemistry, 10 pellets from ACP clones were digested in 8 M urea under reducing conditions. Total protein concentration was quantified through a BCA Assay (Thermo), and equal quantities were loaded onto an 8 % polyacrylamide gel. Gels were transferred to polyvinylidene difluoride (PVDF) membrane, blocked with 2 % nonfat milk, and probed for type X collagen for 1 h at room temperature with a primary antibody to the helical domain (X53, HRP-conjugated mouse monoclonal antibody, 1:5000). The X53 antibody was characterized as described by Girkontaite et al. [41], and full-length collagen type X stably expressed in HEK293 cells was affinity purified as outlined in Wagner et al. [42]. Both reagents were kindly provided by Greg Lunstrum of the Shriners Hospital for Children, Portland, OR, USA. As a loading control, the membrane was probed with a primary antibody to GAPDH (MAB374 mouse monoclonal antibody, 1:300; EMD Millipore, Darmstadt, Germany) for 1 h at room temperature and subsequently probed with HRP-conjugated goat anti-mouse (sc2005, 1:5000; Santa Cruz Biotechnology, Dallas, TX, USA) for 45 min at room temperature. HRP-conjugated antibodies were reacted with Western Lighting Plus ECL (Perkin Elmer, Waltham, MA, USA) chemiluminescence HRP substrate and visualized with a c-Digit blot scanner (LI-COR, Lincoln, NE, USA).

### Statistical analysis

All statistical analyses were performed using GraphPad Prism v6.0 (GraphPad, La Jolla, CA, USA). Normality for each condition within a single cell type was assessed using a D’Agostino–Pearson omnibus K2 test, with normally distributed groups not meeting significance of *p* < 0.05 for distribution other than Gaussian. Comparison of GAG content and gene expression between physioxia and hyperoxia within a given group was assessed using a paired *t* test for normal data and a Wilcoxon matched-pairs signed rank test for nonnormal data, with significance set at *p* < 0.05. Comparison of GAG content between normally distributed groups was performed with an unpaired *t* test, with significance set at *p* < 0.05. Mean and standard deviation for fold-change gene expression was calculated as physioxia relative to hyperoxia for each group.

## Results

### Stem cell populations vary in their response to physioxia during chondrogenic differentiation

**Fig. 1:**
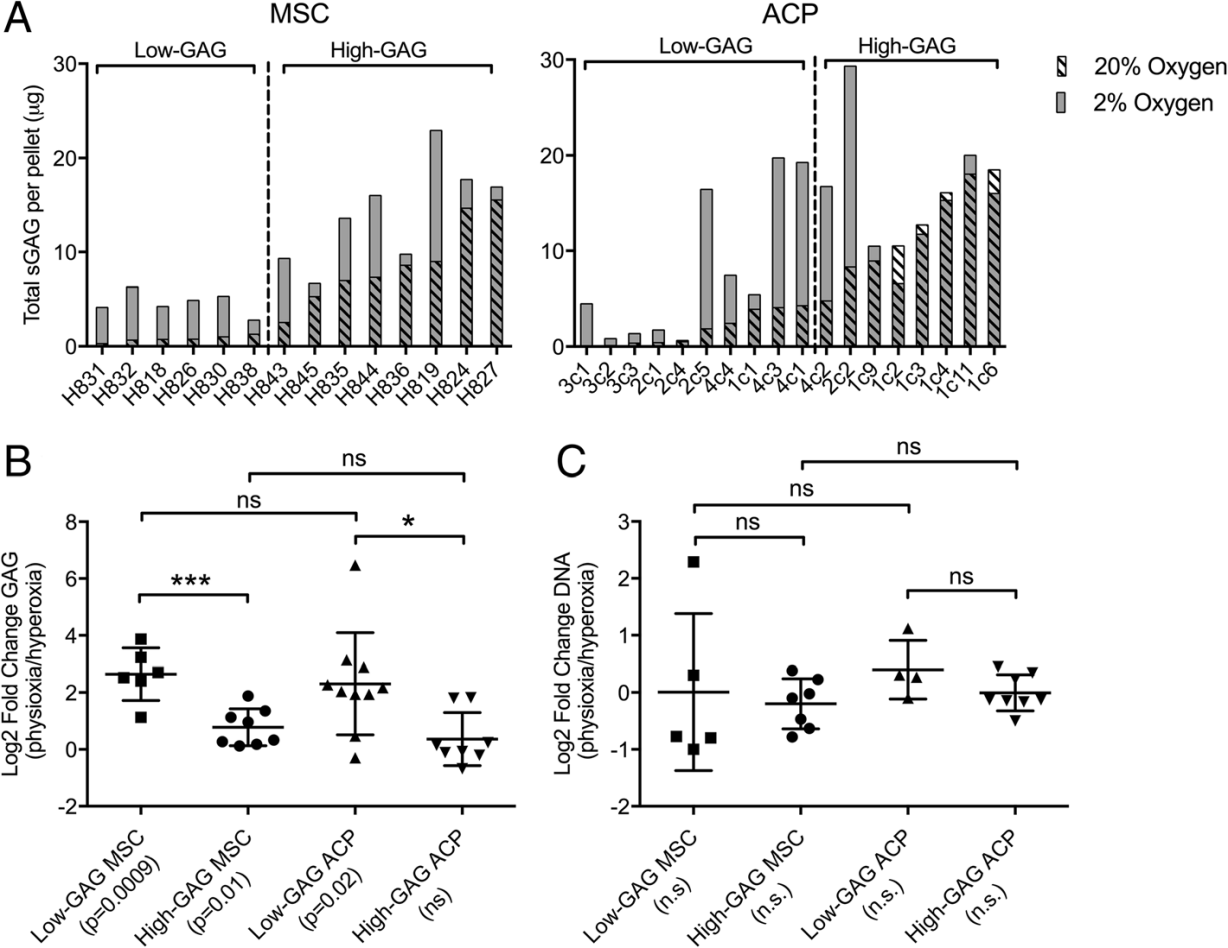
**a** Total glycosaminoglycan (*GAG*) production per pellet for each MSC preparation and ACP clone indicated variation among human donors. A threshold (*dashed line*) defined as two standard deviations from the total GAG production during pellet chondrogenesis for healthy human chondrocytes was set for each cell type, and low-GAG and high-GAG groups were categorized. **b** Mean (± SD) fold-change GAG production in physioxia relative to hyperoxia was significant for all groups other than high-GAG ACPs, and a significant difference existed between the fold-change for low-GAG and high-GAG groups of each cell type. **c** Mean (± SD) fold-change DNA content in physioxia relative to hyperoxia was no different between oxygen level, GAG level, nor cell type. Statistical significance defined as *p < 0.05, ***p < 0.001. *ACP* articular cartilage progenitor, *MSC* mesenchymal stem cell

**Fig. 2:**
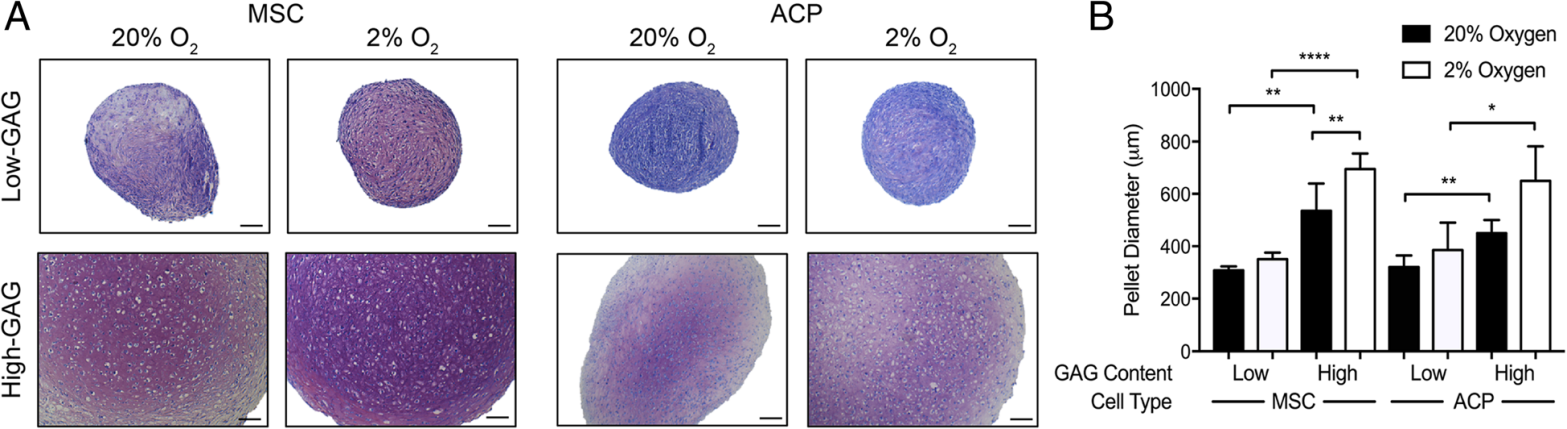
**a** Representative toluidine blue stain for total proteoglycans demonstrates smaller pellets with less metachromasia for low-GAG MSC preparations and ACP clones in both hyperoxia and physioxia relative to paired high-GAG MSC preparations and ACP clones at the respective oxygen levels. Images were acquired with bright-field microscopy, *scale bars* = 100 μm. **b** Measurement of pellet diameter revealed a statistically significant difference in pellet size between both MSCs and ACPs of high or low chondrogenicity and between high-GAG MSCs at physioxia or hyperoxia. Statistical significance defined as *p < 0.05, **p < 0.01, ****p < 0.0001. *ACP* articular cartilage progenitor, *GAG* glycosaminoglycan, *MSC* mesenchymal stem cell

Quantification of total GAG per pellet as a readout for overall chondrogenic differentiation revealed that culture in physioxia significantly favored chondrogenic extracellular matrix anabolism when compared with culture in hyperoxia (*p* = 0.0002 for MSCs, *p* = 0.0124 for ACPs). There existed a wide range of both baseline GAG content in hyperoxia and fold-change GAG content from hyperoxia to physioxia across individual populations of cells (Fig. 1a). Each biologic replicate of MSCs and ACPs was categorized as high-GAG or low-GAG based on a threshold defined by their total GAG production in hyperoxia relative to that of pellet cultures of healthy human articular chondrocytes in the same conditions (Additional file 1: Figure S1). Specifically, cell populations that produced GAGs within two standard deviations of human chondrocyte pellets were considered high-GAG cells, and those below the two standard deviation threshold were considered low-GAG cells. While physioxic culture increased GAG production across all MSC preps and the majority of ACP clones, physioxia was of greater benefit to biologic replicates that exhibited very low GAG production at baseline in hyperoxia, driving a greater fold-change than for clones that started with high GAG production and chondrogenic capacity in hyperoxia (Fig. 1b). There were no statistically significant differences in pellet DNA content between oxygen levels, GAG levels, or cell types, indicating that differences in GAG content were not due to cell proliferation or death (Fig. 1c). Even with a significantly higher fold-induction, the pellets of low-GAG cell preparations of both cell types were still poorly chondrogenic in comparison with matched high-GAG pellets. This can also be discerned easily in the qualitative analysis of proteoglycan and glycosaminoglycan production through toluidine blue staining and quantitative analysis of pellet size; low-GAG pellets of each cell type were significantly smaller in pellet diameter with much less metachromasia than high-GAG pellets of the same cell type (Fig. 2).

### Effect of physioxia on gene expression corresponds with intrinsic chondrogenic capacity

**Fig. 3:**
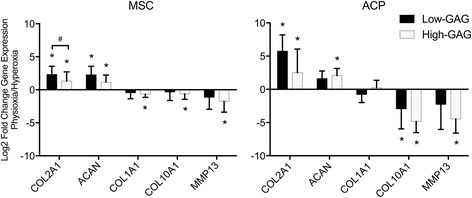
Gene expression analysis for fold-change of chondrogenic markers of the articular cartilage phenotype (*COL2A1*, *ACAN*), the fibrocartilaginous phenotype (*COL1A1*), and the hypertrophic phenotype (*COL10A1*, *MMP13*) demonstrates varied chondrogenic responses by high-GAG and low-GAG groups of each cell type, MSCs and ACPs, during pellet culture in physioxic relative to hyperoxic conditions. Data are mean ± standard deviation of fold-change for each group (*n* = 6–10). Statistical significance defined as *p < 0.05 by a paired or unpaired *t* test where appropriate. # *p* < 0.05 between low and high GAG pellets. *ACP* articular cartilage progenitor, *GAG* glycosaminoglycan, *MSC* mesenchymal stem cell

All cell preparations and clones were evaluated for chondrogenic markers, including those of the articular chondrocyte (*COL2A1*, *ACAN*), the fibrochondrocyte (*COL1A1*), and the hypertrophic chondrocyte (*COL10A1*, *MMP13*) phenotypes. Physioxic culture significantly upregulated *COL2A1* and *ACAN* in low-GAG preparations from both cell types (Fig. 3). However, among low-GAG cells, only ACPs demonstrated significant downregulation of *COL10A1* while MSCs did not downregulate either marker of the hypertrophic phenotype, *COL10A1* or *MMP13*. Physioxic culture of the high-GAG cells demonstrated significant upregulation of *COL2A1* and *ACAN* for both ACPs and MSCs, with a corresponding significant downregulation of *COL10A1* and MMP13 relative to culture in hyperoxia. Only high-GAG MSCs demonstrated a significant downregulation of *COL1A1* gene expression.

### Oxygen-dependent genes are differentially expressed between groups of low and high chondrogenicity

**Fig. 4:**
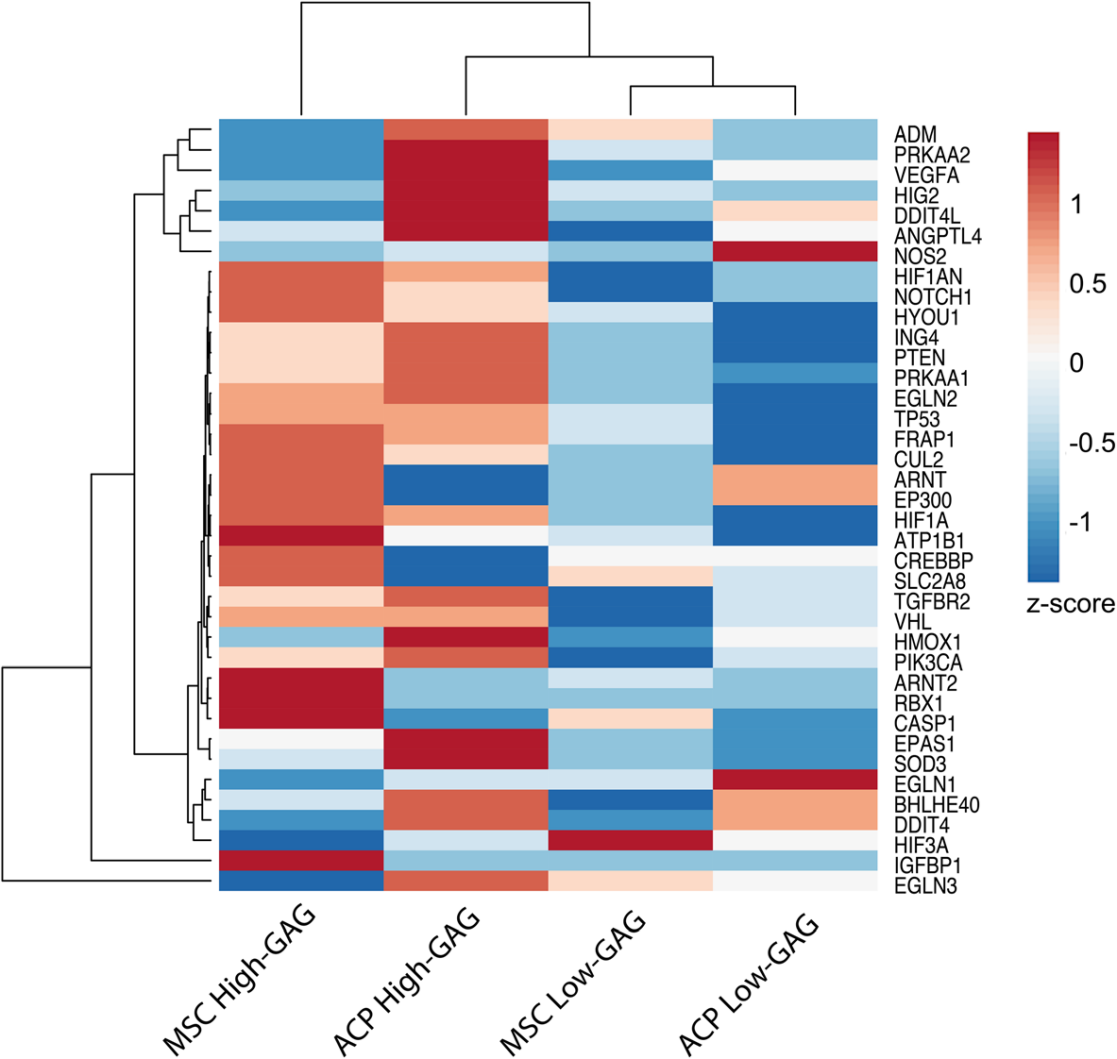
Clustering of oxygen-dependent gene expression based on *z*-score demonstrates that groups of MSCs and ACPs are more similar between GAG level than within cell type in response to culture in physioxia relative to culture in hyperoxia. *ACP* articular cartilage progenitor, *GAG* glycosaminoglycan, *MSC* mesenchymal stem cell

When evaluated for a panel of oxygen-responsive genes, high-GAG MSCs and ACPs displayed a different expression pattern to the matched low-GAG group for each cell type. Based on cluster analysis, cell groups were more closely related based on GAG production and intrinsic chondrogenicity than on cell type; low-GAG MSCs and ACPs clustered together, which then clustered with high-GAG ACPs, and finally with high-GAG MSCs (Fig. 4). When evaluated as the relative fold-change from hyperoxia to physioxia (Additional file 2: Figure S2), high-GAG MSCs upregulated a majority (80 %) of oxygen-responsive genes, including those of the HIF regulatory and TGF-β signaling pathways, both of which modulate chondrogenic differentiation. These cells also either upregulated or did not change oxygen-responsive genes that regulate other biological processes, grouped by gene ontology, including cell cycle control, metabolism, and angiogenesis; many genes were involved in two or more processes. In contrast, low-GAG MSCs downregulated 74 % of these same genes across all groupings. Both high-GAG and low-GAG ACPs downregulated a majority of these oxygen-dependent genes, and the response between groups was similar in magnitude as well as direction for most genes.

### Physioxia promotes the articular chondrocyte phenotype in highly chondrogenic cells

**Fig. 5:**
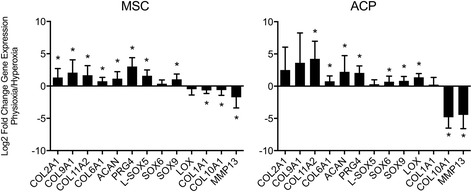
Gene expression analysis for fold-change of chondrogenic markers in physioxia relative to hyperoxia demonstrates that high-GAG groups of both MSCs and ACPs are highly responsive to oxygen level and upregulate a majority of genes representative of the articular cartilage phenotype in low-oxygen environments. Data are mean ± standard deviation of fold-change in gene expression for each group (*n* = 8). Statistical significance defined as *p < 0.05 by a paired *t* test for normal data and a Wilcoxon matched-pairs signed rank test for nonnormal data. *ACP* articular cartilage progenitor, *MSC* mesenchymal stem cell

High-GAG cells of both MSCs and ACPs demonstrated significant upregulation of *COL2A1*, *COL11A2*, *COL6A1*, *ACAN*, *PRG4*, and *SOX9* and downregulation of *COL10A1* and *MMP13* in physioxia relative to hyperoxia (Fig. 5, Additional file 3: Table S1). High-GAG MSC preparations also demonstrated significant upregulation of *COL9A1* and *L-SOX5* and downregulation of *COL1A1* in physioxia. High-GAG ACP clones further demonstrated significant upregulation of *SOX6* and *LOX. COL9A1* was undetectable in five of eight high-GAG ACP clones cultured in hyperoxia but present at detectable levels in all clones cultured in physioxia.

### Physioxia promotes collagen protein expression representative of articular cartilage

**Fig. 6:**
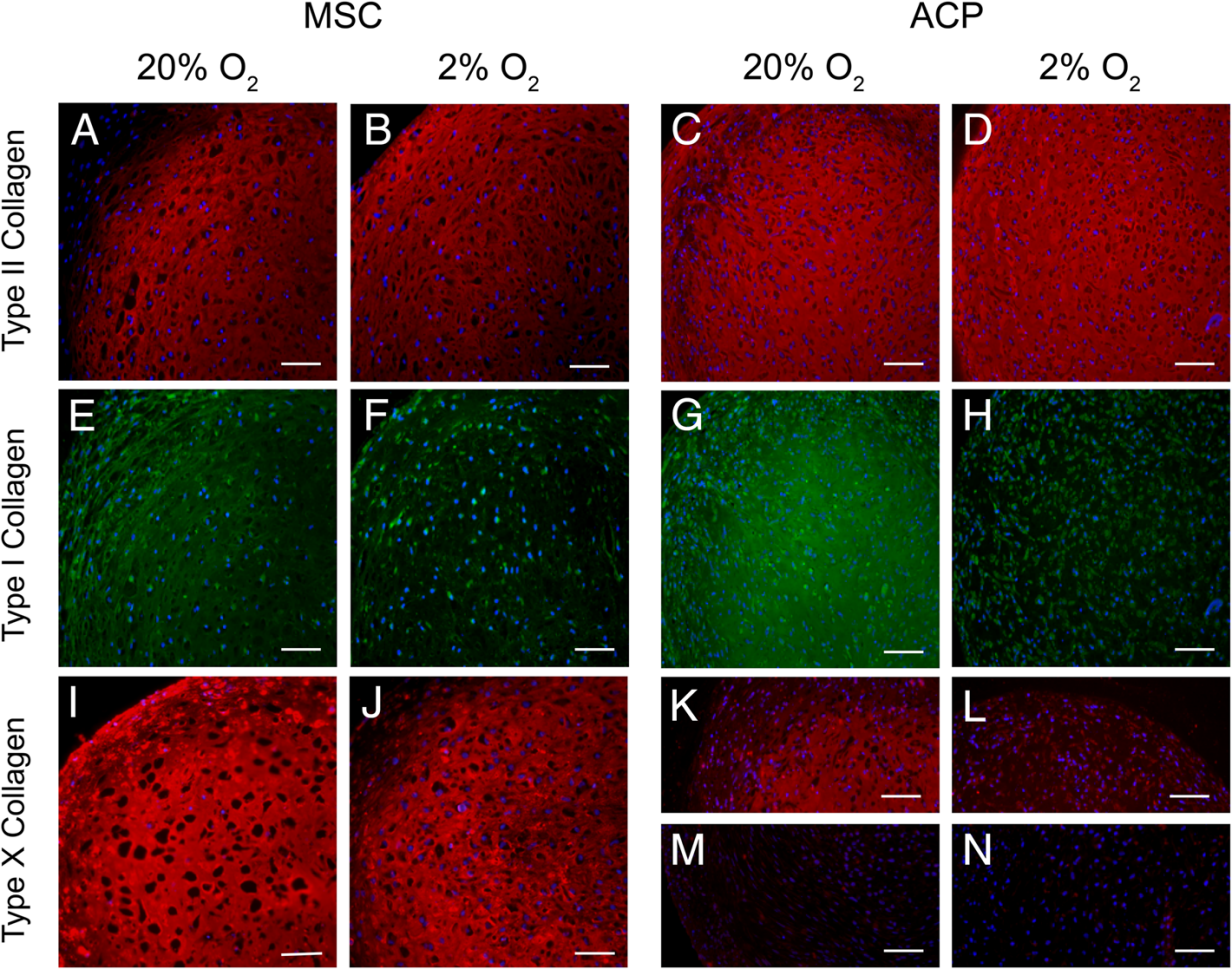
Representative immunohistochemistry demonstrates that cartilage pellets cultured in hyperoxia (20 % oxygen) and physioxia (2 % oxygen) exhibit oxygen-dependent expression of extracellular collagen protein, including type II collagen (**a**–**d**) of the articular cartilage phenotype, type I collagen (**e**–**h**) of the fibrocartilaginous phenotype, and type X collagen (**i**–**n**) of the hypertrophic phenotype. Type X collagen expression was variable among ACP clones differentiated in hyperoxia (**k**, **m**). Nuclei were counterstained with DAPI, and composite images of collagen staining and nuclei were digitally merged using ImageJ software (National Institutes of Health, Bethesda, MD, USA). Negative controls with isotype-matched antibodies were used for background correction (images not shown). *Scale bars* = 100 μm. *ACP* articular cartilage progenitor, *MSC* mesenchymal stem cell

Culture of high-GAG MSCs and ACPs in both physioxic and hyperoxic environments generated cartilage pellets with robust type II collagen expression throughout (Fig. 6). MSCs cultured in hyperoxia exhibited highest type I collagen expression in the outer region of the pellet, whereas MSCs cultured in physioxia demonstrated less staining of extracellular type I collagen, consistent with significant downregulation of *COL1A1*. Similarly, high-GAG ACPs demonstrated a reduction in extracellular type I collagen in physioxia compared with hyperoxia. Type X collagen remained consistently high in all high-GAG MSC preparations evaluated, regardless of oxygen tension. Conversely, high-GAG ACPs cultured in hyperoxia demonstrated variable type X collagen protein between clones, ranging from abundant to absent. Significantly, type X collagen protein expression was undetectable in the extracellular matrix of all clones cultured in physioxia. Western blots of pellet extracts with antibodies to type X collagen generated consistent results with little to no detectable protein in ACP clones at physioxia (Additional file 4: Figure S3).

## Discussion

From our results, we can state that physioxia promotes the stable articular cartilage phenotype at both the gene and protein levels during chondrogenic differentiation of ACPs in three-dimensional culture*.* MSCs demonstrated a similar response with respect to gene expression; however, type X collagen expression persisted in the physioxic environment. The magnitude of anabolic response to culture in physioxia was inversely related to the intrinsic chondrogenic potential in hyperoxia for both cell types. To account for differences in the chondrogenic capacity of stem cells at baseline, we divided the cell populations based on total GAG production. Those that produced low quantities of GAGs in a hyperoxic environment gained significant benefit from culture in physioxia but not enough to elaborate the matrix to the levels produced by cells with high intrinsic chondrogenic capacity. In contrast, cells that produced high quantities of GAGs in hyperoxia did not respond as robustly to lowered oxygen; these cells may be closer to reaching their maximum rate of proteoglycan synthesis that oxygen-related mechanisms are unable to further increase. The differences in GAG production between replicates and clones was due to enhanced anabolism and not cell proliferation or death, as the DNA content was not different between these groups. While we did not directly measure the influence of donor age or gender on variation in chondrogenic capacity, the wide range across ACP clones and replicates, which were all derived from young males, demonstrates the extent of variation in human cells; these cells showed as wide a range in chondrogenicity as MSCs, which were derived from older male and female donors.

For low-GAG MSCs and ACPs, a significant increase in markers of the articular cartilage tissue phenotype, including GAG content and *COL2A1* and *ACAN* gene expression, was not complemented by significantly decreased expression of both hypertrophic genes, *COL10A1* and *MMP13*. Despite a substantial fold-change in chondrogenic markers from hyperoxia to physioxia, these cells produced consistently smaller and more fibrous pellets in comparison with the high-GAG producers of the same cell type. In contrast, high-GAG MSC preparations and ACP clones significantly downregulated hypertrophic markers with culture in physioxia, demonstrating a distinct phenotypic difference between groups of cells of varied chondrogenic differentiation potential at baseline. Differences in gene expression between high-GAG and low-GAG preparations were not limited to genes of chondrogenic differentiation; at low oxygen tension, high-GAG MSCs upregulated canonical hypoxia-responsive genes—including those of HIF regulation, constituents of TGF-β and IGF signaling, and cell cycle regulators—relative to hyperoxic culture. In contrast, low-GAG MSCs were most similar to ACPs of low chondrogenicity, because both downregulated or did not change a majority of these oxygen-responsive genes in response to culture in physioxia. Expression of oxygen-responsive genes for high-GAG ACPs clustered with high-GAG MSCs and the grouping of low-GAG cell populations, indicating a mid-range response to physioxia in comparison with high-GAG MSCs, which were most responsive to physioxia. The differences between MSCs of high and low chondrogenicity in both direction and magnitude may be due to the cellular heterogeneity within a given preparation, which represents the total population of plastic adherent mononuclear cells from a bone marrow aspirate. Poorly chondrogenic cells expanded from this material may originate from a different population of cells than highly chondrogenic cells, and their dramatically different response across oxygen-dependent genes indicates they are indeed very different cells, not simply different in their ability to express cartilage genes and elaborate matrix. ACPs, on the contrary, are selected based on expression of specific integrins from a comparatively homogeneous initial pool of cells. These cells correspondingly are overall much more similar in their response to changes in oxygen, regardless of their ability to produce matrix-rich tissue. Taken together, our findings regarding both chondrogenic and oxygen-dependent gene expression indicate that low-GAG MSCs may be intrinsically limited in their overall response to altered oxygen levels.

Discrepancies in the literature regarding MSC hypertrophy during chondrogenic differentiation in lower oxygen may be due to inclusion of preparations of low chondrogenic capacity, and the present results demonstrate wide variation not previously taken into consideration for either cell type when examining their responses to alterations in oxygen level. An earlier study of MSC chondrogenesis in lowered oxygen tension reported a significant upregulation of *COL10A1* for cells expanded without FGF-2 [17], which is known to aid retention of chondrogenic capacity in these cells [19–21]. Our later studies demonstrated a significant downregulation of hypertrophic genes when differentiating MSCs of previously defined high chondrogenic capacity in lowered oxygen [12]. Other recent studies have also reported downregulation of hypertrophic genes during MSC differentiation in lowered oxygen tension [9–11, 13]. Adesida et al. [9] reported that MSCs expanded and differentiated in pellet culture at low oxygen tension significantly downregulate *COL10A1* in comparison with hyperoxic cultured MSCs; however, Bornes et al. [16] from the same laboratory found no change in *COL10A1* expression for ovine MSCs cultured at low and high oxygen in a scaffold-based culture system. Studies of a larger set of human MSC preparations found no change in *COL10A1* expression with culture in physioxia when compared with hyperoxia, but these studies did not characterize individual preparations for baseline chondrogenicity [14, 15]. Finally, Duval et al. [11] showed that *COL10A1* expression is downregulated in the presence of HIF-1α and upregulated with blockade of this protein; however, mechanisms underlying HIF activity and *COL10A1* expression remain unclear [12, 43–45]. Based on our investigation, we suggest that the variation in the reported results for the hypertrophic response of MSCs in lowered oxygen tension may be due to the disparity of the intrinsic chondrogenic capacity of these cells at baseline: highly chondrogenic cells significantly downregulate genes of hypertrophy, and cells of lower chondrogenic potential are much less responsive.

We reasoned that that cells of low chondrogenic capacity, such as the low-GAG MSC preparations and ACP clones, are unsuitable candidates for articular cartilage tissue engineering application and focused on the responses of high-GAG cells to culture in physioxia to further characterize the effects of oxygen on the articular chondrocyte phenotype. In low-oxygen conditions, HIF-1α and HIF-2α are considered prochondrogenic through induction of *SOX9*, the gene coding for the master transcriptional regulator of chondrogenesis, SOX9, that in turn promotes *COL2A1* and *ACAN* gene expression to drive extracellular matrix anabolism of type II collagen and aggrecan, respectively [46, 47]. As expected, both MSCs and ACPs significantly upregulated *SOX9* in response to lowered oxygen, and MSCs significantly upregulated *L-*SOX5, the gene for a complementary chondrogenic transcription factor, while ACPs significantly upregulated *SOX6*. HIF-1α has also been reported to activate transcription of *LOX*—the gene coding for lysyl oxidase, which is an enzyme that catalyzes collagen crosslinking in articular cartilage through a hypoxia-responsive element (HRE) [48], but only ACPs demonstrated a significant upregulation of *LOX*. Conclusions regarding the regulation of *PRG4*, the gene for lubricin, in response to alterations in oxygen vary in the literature, even though *PRG4* also contains an HRE in the promoter region [45, 49, 50]. We have shown here that highly chondrogenic cells upregulated *PRG4* gene expression in physioxia. *COL9A1*, *COL11A1*, and *COL6A1*, the genes for type IX, type XI, and type VI collagen respectively, were upregulated in both cell types, indicating that the collagen network is extensively influenced by lowered oxygen tension toward the articular phenotype. Only MSCs demonstrated a significant downregulation of *COL1A1*; however, both MSCs and ACPs demonstrated a reduction of extracellular type I collagen protein with culture in physioxia, indicating the influence of oxygen on posttranslational modifications of the collagen network formed by these cells. Specifically, proline hydroxylation is a necessary step for successful collagen secretion, and this oxygen-dependent enzymatic reaction may be attenuated in low-oxygen environments [51]. When oxygen is limited, chondrogenic cells may preferentially secrete type II over type I collagen, as *COL2A1* is upregulated in an oxygen-dependent manner through HIF regulation [47]. Consistent with this hypothesis, our group has shown that articular chondrocytes also downregulate type I collagen at both the gene and protein levels in response to lowered oxygen tension [8]. These findings indicate that culture in low oxygen favors the articular chondrocyte phenotype over the fibrocartilage phenotype for multiple cell types.

The findings in this study are in agreement with recent reports on the beneficial effect of low oxygen in reducing markers of hypertrophy at the gene level in MSCs [9, 12, 13], at least for our preparations of high chondrogenic capacity. Although highly chondrogenic MSC preparations significantly downregulated hypertrophic genes in physioxia, these cells still produced a matrix rich in type X collagen protein. Prior studies that reported a decrease in *COL10A1* for MSCs differentiated in low oxygen did not evaluate type X collagen protein expression [9, 12, 13]. Leijten et al. [13] reported recently that low-oxygen culture of MSCs abrogated expression of typical hypertrophic markers at the mRNA level and diminished subsequent vascular invasion and ossification of implanted constructs. While this may indeed be a benefit of low-oxygen culture, we show that such preconditioning still likely results in tissue rich in type X collagen protein and this may have consequences unrelated to endochondral ossification. Ultimately, type X collagen establishes a tissue phenotype with increased stiffness that is not suited for articular cartilage repair [52]. Culture of MSC-derived cartilage at physioxia does not overcome the long-noted challenge of controlling MSC differentiation to avoid creating the hypertrophic phenotype.

In the initial characterization of chondrogenic differentiation of ACPs in hyperoxia, Williams et al. [30] did not find clones that produced detectable type X collagen at the protein level. In contrast, we found that some ACP clones produced abundant type X collagen in the hyperoxic environment. This notwithstanding, none of the clones produced detectable type X collagen in the physioxic environment. While both MSCs and ACPs of high chondrogenicity express *COL10A1*, differences in protein expression between physioxia and hyperoxia indicate that these two stem cell populations may have different translational control over this gene. The difference in translational control will be the subject of future studies. Regardless of the mechanism, ACP culture in physioxia consistently promotes a favorable tissue phenotype when compared with MSC-derived tissue.

## Conclusions

The variation in chondrogenic capacity among stem/progenitor cell populations from adult human tissues is an important consideration in tissue engineering. Our work suggests that laboratories ought to perform initial assays to characterize individual populations of human-derived stem cells, because variation in intrinsic chondrogenicity will influence experimental results, especially for studies with a small sample size. Although the impact of intrinsic chondrogenicity was not evaluated in vivo, we further reason that individual stem cell populations intended for allogeneic or autologous tissue repair should be characterized in vitro to identify highly chondrogenic cells prior to application in tissue-engineered therapies. We have shown that the chondrogenic capacity of stem cells influences the cell’s response to culture in a physiologic low-oxygen environment: in physioxia, both MSCs and ACPs of high chondrogenicity demonstrate upregulation of the articular chondrocyte phenotype and downregulation of the hypertrophic phenotype. Only ACPs, however, demonstrate a consistent attenuation of type X collagen in the physioxic environment, and these cells may overcome historical challenges of MSC hypertrophy in tissue engineering applications.

## Additional files

Additional file 1: Figure S1 showing total glycosaminoglycan (*GAG*) production per pellet for healthy human chondrocytes cultured at densities of 100,000 or 50,000 cells per pellet over 14 days of chondrogenic differentiation. Two standard deviations below the mean of each group (*dotted line*) was used to define the threshold for grouping MSC and ACP preparations based on proteoglycan production for the matched cell density. (TIFF 491 kb)
Additional file 2: Figure S2 showing fold-change gene expression from hyperoxia to physioxia across an array of oxygen-dependent genes, and demonstrates differential expression patterns between high-GAG and low-GAG groups of each cell type, MSCs and ACPs. Genes labeled in *green* were upregulated in physioxia relative to hyperoxia, and genes labeled in *red* were downregulated in physioxia relative to hyperoxia. Genes were categorized according to associations of gene ontology for biologic processes based on associations and clusters in the STRING database. (TIF 8318 kb)
Additional file 3: Table S1 presenting gene expression. (PDF 72 kb)
Additional file 4: Figure S3 showing western blots to detect type X collagen in total protein lysates from ACP pellets cultured for 14 days, and demonstrates that ACPs either lack type X collagen expression in all conditions or reduce expression with culture in physioxia relative to culture in hyperoxia. (TIF 4269 kb)

## Abbreviations

ACP: Articular cartilage progenitor
BSA: Bovine serum albumin
DAPI: 4′,6-diamidino-2-phenylindole
DMEM: Dulbecco’s modified Eagle medium
DMMB: 1,9-Dimethylmethylene Blue
FBS: Fetal bovine serum
FGF-2: Fibroblast growth factor 2
GAG: Glycosaminoglycan
HIF: Hypoxia inducible factor
HRP: Horseradish peroxidase
MSC: Mesenchymal stem cell
NGS: Normal goat serum
NHS: National Health Service
OHSU: Oregon Health & Science University
PBS: Phosphate-buffered saline
STRING: Search Tool for the Retrieval of Interacting Genes/Proteins
TGF-β: Transforming growth factor beta

## References

[1] Johnstone B, Hering T, Caplan AI, Goldberg VM, Yoo JU (1998). In vitro chondrogenesis of bone marrow-derived mesenchymal progenitor cells. Exp Cell Res..

[2] Johnstone B, Alini M, Cucchiarini M, Dodge GR, Eglin D, Guilak F (2013). Tissue engineering for articular cartilage repair—the state of the art. Eur Cells Mater..

[3] Carreau A, Hafny-Rahbi BE, Matejuk A, Grillon C, Kieda C (2011). Why is the partial oxygen pressure of human tissues a crucial parameter? Small molecules and hypoxia. J Cell Mol Med..

[4] Lund-Olesen K (1970). Oxygen tension in synovial fluids. Arthritis Rheum..

[5] Brighton CT, Heppenstall RB (1971). Oxygen tension in zones of the epiphyseal plate, the metaphysis and diaphysis. An in vitro and in vivo study in rats and rabbits. J Bone Joint Surg Am.

[6] Lafont JE (2010). Lack of oxygen in articular cartilage: consequences for chondrocyte biology. Int J Exp Pathol..

[7] Murphy CL, Thomas BL, Vaghjiani RJ, Lafont JE. HIF-mediated articular chondrocyte function: prospects for cartilage repair. Arthritis Res Ther. 2009:1–7 10.1186/ar2574 PMC2688225 19232075

[8] Markway BD, Cho H, Johnstone B (2013). Hypoxia promotes redifferentiation and suppresses markers of hypertrophy and degeneration in both healthy and osteoarthritic chondrocytes. Arthritis Res Ther..

[9] Adesida AB, Mulet-Sierra A, Jomha NM (2012). Hypoxia mediated isolation and expansion enhances the chondrogenic capacity of bone marrow mesenchymal stromal cells. Stem Cell Res Ther..

[10] Gawlitta D, van Rijen MHP, Schrijver EJM, Alblas J, Dhert WJA (2012). Hypoxia impedes hypertrophic chondrogenesis of human multipotent stromal cells. Tissue Eng Pt A..

[11] Duval E, Baugé C, Andriamanalijaona R, Bénateau H, Leclercq S, Dutoit S (2012). Molecular mechanism of hypoxia-induced chondrogenesis and its application in in vivo cartilage tissue engineering. Biomaterials..

[12] Markway BD, Cho H, Zilberman-Rudenko J, Holden P, McAlinden A, Johnstone B (2015). Hypoxia-inducible factor 3-alpha expression is associated with the stable chondrocyte phenotype. J Orthop Res..

[13] Leijten J, Georgi N, Moreira Teixeira L, van Blitterswijk CA, Post JN, Karperien M (2014). Metabolic programming of mesenchymal stromal cells by oxygen tension directs chondrogenic cell fate. Proc Natl Acad Sci U S A..

[14] Felka T, SchAfer R, Schewe B, Benz K, Aicher WK (2009). Hypoxia reduces the inhibitory effect of IL-1beta on chondrogenic differentiation of FCS-free expanded MSC. Osteoarthr Cartilage..

[15] Muller J, Benz K, Ahlers M, Gaissmaier C, Mollenhauer J (2011). Hypoxic conditions during expansion culture prime human mesenchymal stromal precursor cells for chondrogenic differentiation in three-dimensional cultures. Cell Transplant..

[16] Bornes TD, Jomha NM, Mulet-Sierra A, Adesida AB. Hypoxic culture of bone marrow-derived mesenchymal stromal stem cells differentially enhances in vitro chondrogenesis within cell-seeded collagen and hyaluronic acid porous scaffolds. Stem Cell Res Ther. 2015:1–18 10.1186/s13287-015-0075-4 PMC4431536 25900045

[17] Markway BD, Tan G-K, Brooke G, Hudson JE, Cooper-White JJ, Doran MR (2010). Enhanced chondrogenic differentiation of human bone marrow-derived mesenchymal stem cells in low oxygen environment micropellet cultures. Cell Transplant..

[18] Meretoja VV, Dahlin RL, Wright S, Kasper FK, Mikos AG (2013). The effect of hypoxia on the chondrogenic differentiation of co-cultured articular chondrocytes and mesenchymal stem cells in scaffolds. Biomaterials..

[19] Ito T, Sawada R, Fujiwara Y, Tsuchiya T (2007). FGF-2 increases osteogenic and chondrogenic differentiation potentials of human mesenchymal stem cells by inactivation of TGF-β signaling. Cytotechnology..

[20] Cheng T, Yang C, Weber N, Kim HT, Kuo AC (2012). Fibroblast growth factor 2 enhances the kinetics of mesenchymal stem cell chondrogenesis. Biochem Bioph Res Commun..

[21] Handorf AM, Li W-J (2011). Fibroblast growth factor-2 primes human mesenchymal stem cells for enhanced chondrogenesis. ONE..

[22] Hayes AJ, MacPherson S, Morrison H, Dowthwaite GP, Archer CW (2001). The development of articular cartilage: evidence for an appositional growth mechanism. Anat Embryol..

[23] Dowthwaite GP, Bishop JC, Redman SN, Khan IM, Rooney P, Evans DJR (2004). The surface of articular cartilage contains a progenitor cell population. J Cell Sci..

[24] Kozhemyakina E, Zhang M, Ionescu A, Ayturk UM, Ono N, Kobayashi A (2015). Identification of a Prg4-expressing articular cartilage progenitor cell population in mice. Arthritis Rheum..

[25] Koelling S, Kruegel J, Irmer M, Path JR, Sadowski B, Miro X (2009). Migratory chondrogenic progenitor cells from repair tissue during the later stages of human osteoarthritis. Stem Cell..

[26] Gerter R, Kruegel J, Miosge N (2012). New insights into cartilage repair—the role of migratory progenitor cells in osteoarthritis. Matrix Biol..

[27] Seol D, McCabe DJ, Choe H, Zheng H, Yu Y, Jang K (2012). Chondrogenic progenitor cells respond to cartilage injury. Arthritis Rheum..

[28] Nelson L, McCarthy HE, Fairclough J, Williams R, Archer CW (2014). Evidence of a viable pool of stem cells within human osteoarthritic cartilage. Cartilage..

[29] Jiang Y, Tuan RS (2014). Origin and function of cartilage stem/progenitor cells in osteoarthritis. Nat Rev Rhematol..

[30] Williams R, Khan IM, Richardson K, Nelson L, McCarthy HE, Analbelsi T (2010). Identification and clonal characterisation of a progenitor cell sub-population in normal human articular cartilage. Plos ONE..

[31] McCarthy HE, Bara JJ, Brakspear K, Singhrao SK, Archer CW (2012). The comparison of equine articular cartilage progenitor cells and bone marrow-derived stromal cells as potential cell sources for cartilage repair in the horse. Vet J..

[32] Frisbie DD, McCarthy HE, Archer CW, Barrett MF, McIlwraith CW (2015). Evaluation of articular cartilage progenitor cells for the repair of articular defects in an equine model. J Bone Joint Surg..

[33] Jayasuriya CT, Chen Q (2015). Potential benefits and limitations of utilizing chondroprogenitors in cell-based cartilage therapy. Connect Tissue Res..

[34] Khan IM, Bishop JC, Gilbert S, Archer CW (2009). Clonal chondroprogenitors maintain telomerase activity and Sox9 expression during extended monolayer culture and retain chondrogenic potential. Osteoarthr Cartilage..

[35] Mareddy S, Dhaliwal N, Crawford R, Xiao Y (2010). Stem cell-related gene expression in clonal populations of mesenchymal stromal cells from bone marrow. Tissue Eng Pt A..

[36] Pilgaard L, Lund P, Duroux M, Fink T, Ulrich-Vinther M, Søballe K (2009). Effect of oxygen concentration, culture format and donor variability on in vitro chondrogenesis of human adipose tissue-derived stem cells. Regen Med..

[37] Katopodi T, Tew SR, Clegg PD, Hardingham TE (2009). The influence of donor and hypoxic conditions on the assembly of cartilage matrix by osteoarthritic human articular chondrocytes on Hyalograft matrices. Biomaterials..

[38] Farrell MJ, Shin JI, Smith LJ, Mauck RL (2015). Functional consequences of glucose and oxygen deprivationon engineered mesenchymal stem cell-based cartilage constructs. Osteoarthr Cartilage..

[39] Russell KC, Phinney DG, Lacey MR, Barrilleaux BL, Meyertholen KE, O’Connor KC (2010). In vitro high-capacity assay to quantify the clonal heterogeneity in trilineage potential of mesenchymal stem cells reveals a complex hierarchy of lineage commitment. Stem Cells..

[40] Russell KC, Lacey MR, Gilliam JK, Tucker HA, Phinney DG, O’Connor KC (2011). Clonal analysis of the proliferation potential of human bone marrow mesenchymal stem cells as a function of potency. Biotechnol Bioeng..

[41] Girkontaite I, Frischholz S, Lammi P, Wagner K, Swoboda B, Aigner T (1996). Immunolocalization of type X collagen in normal fetal and adult osteoarthritic cartilage with monoclonal anti-bodies. Matrix Biol..

[42] Wagner K, Poschl E, Turnay J, Baik J-M, Pihlajaniemi T, Frischholz S, et al. Coexpression of α and β subunits of prolyl 4-hydroxylase stabilizes the triple helix of recombinant human type X collagen. Biochem. J. 2000:907–11 PMC1221533 11104702

[43] Saito T, Fukai A, Mabuchi A, Ikeda T, Yano F, Ohba S (2010). Transcriptional regulation of endochondral ossification by HIF-2α during skeletal growth and osteoarthritis development. Nat Med..

[44] Yang S, Kim J, Ryu J-H, Oh H, Chun C-H, Kim BJ (2010). Hypoxia-inducible factor-2α is a catabolic regulator of osteoarthritic cartilage destruction. Nat Med..

[45] Ruan MZC, Erez A, Guse K, Dawson B, Bertin T, Chen Y (2013). Proteoglycan 4 expression protects against the development of osteoarthritis. Sci Trans Med.

[46] Lafont JE, Talma S, Hopfgarten C, Murphy CL (2007). Hypoxia promotes the differentiated human articular chondrocyte phenotype through SOX9-dependent and -independent pathways. J Biol Chem..

[47] Duval E, Leclercq S, Elissalde J-M, Demoor M, Galéra P, Boumédiene K (2009). Hypoxia-inducible factor 1alpha inhibits the fibroblast-like markers type I and type III collagen during hypoxia-induced chondrocyte redifferentiation. Arthritis Rheum..

[48] Erler JT, Bennewith KL, Nicolau M, Dornhöfer N, Kong C, Le Q-T (2006). Lysyl oxidase is essential for hypoxia-induced metastasis. Nature..

[49] Hatta T, Kishimoto KN, Okuno H, Itoi E (2014). Oxygen tension affects lubricin expression in chondrocytes. Tissue Eng Pt A..

[50] Mhanna R, Öztürk E, Schlink P, Zenobi-Wong M (2013). Probing the microenvironmental conditions for induction of superficial zone protein expression. Osteoarthr Cartilage..

[51] Bentovim L, Amarilio R, Zelzer E (2012). HIF1α is a central regulator of collagen hydroxylation and secretion under hypoxia during bone development. Development..

[52] Aspden RM (1994). Fibre reinforcing by collagen in cartilage and soft connective tissues. Proc Biol Soc..

