# Supplementary figures and images for: Responses to altered oxygen tension are distinct between human stem cells of high and low chondrogenic capacity
Source: Stem Cell Res Ther. 2016 Oct 20;7:154. doi: 10.1186/s13287-016-0419-8 (PMC5073443; doi:10.1186/s13287-016-0419-8)

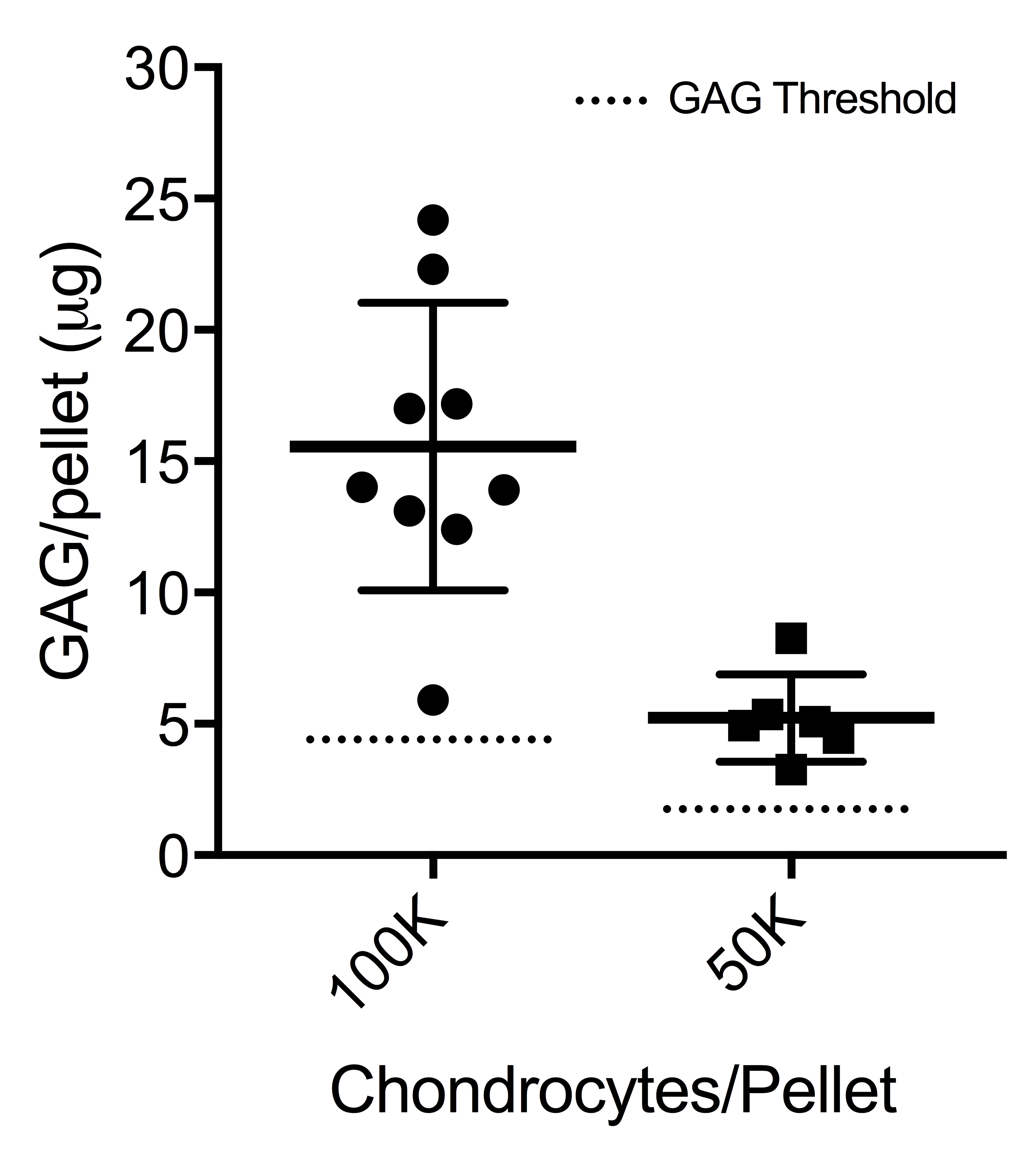

Supplement: Additional file 1: — Figure S1 showing total glycosaminoglycan (GAG) production per pellet for healthy human chondrocytes cultured at densities of 100,000 or 50,000 cells per pellet over 14 days of chondrogenic differentiation. Two standard deviations below the mean of each group (dotted line) was used to define the threshold for grouping MSC and ACP preparations based on proteoglycan production for the matched cell density. (TIFF 491 kb) [file 13287_2016_419_MOESM1_ESM.tiff]

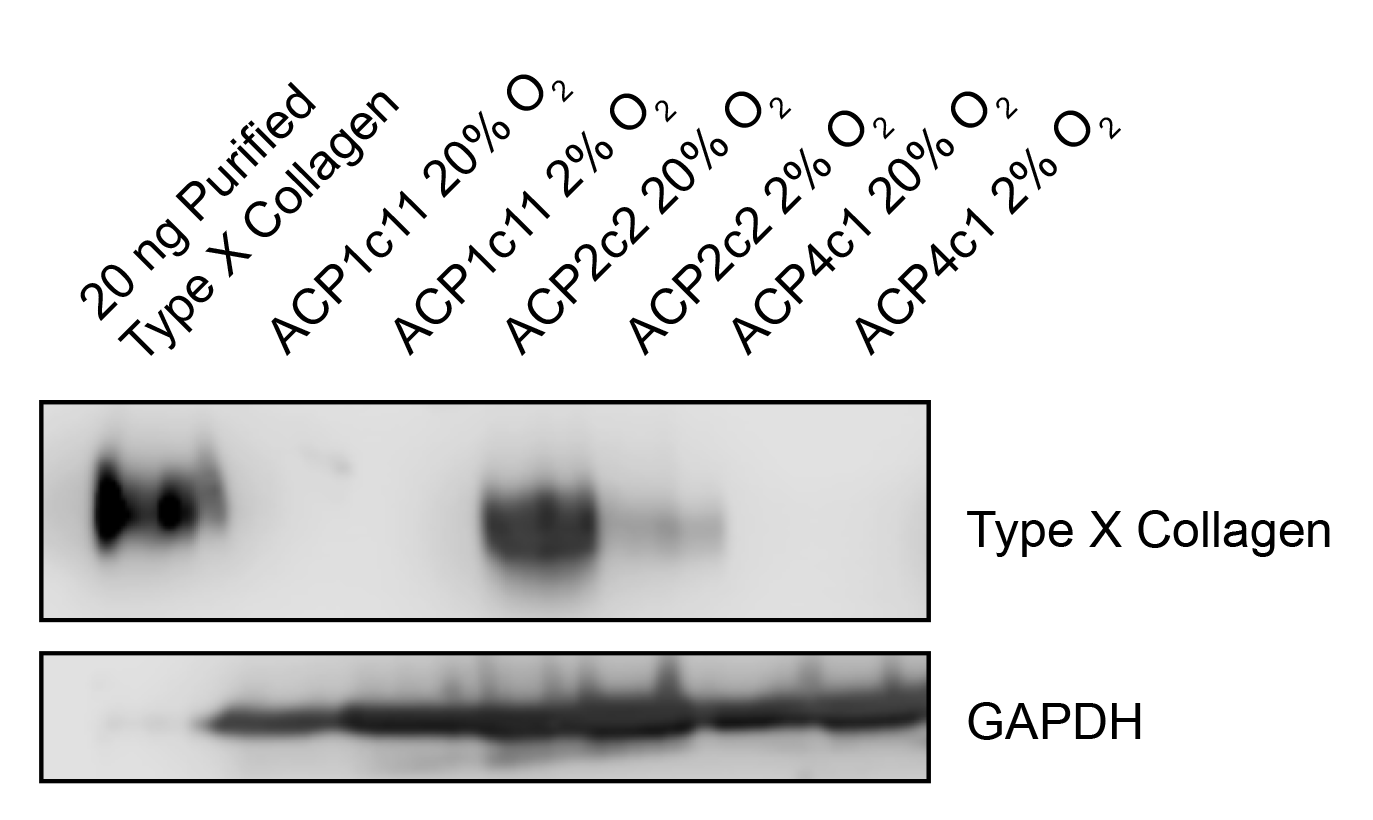

Supplement: Additional file 4: — Figure S3 showing western blots to detect type X collagen in total protein lysates from ACP pellets cultured for 14 days, and demonstrates that ACPs either lack type X collagen expression in all conditions or reduce expression with culture in physioxia relative to culture in hyperoxia. (TIF 4269 kb) [file 13287_2016_419_MOESM4_ESM.tif]
