# Supplementary material for: Responses to altered oxygen tension are distinct between human stem cells of high and low chondrogenic capacity
Source: Stem Cell Res Ther. 2016 Oct 20;7:154. doi: 10.1186/s13287-016-0419-8 (PMC5073443; doi:10.1186/s13287-016-0419-8)
Supplement: Additional file 3: — Table S1 presenting gene expression. (PDF 72 kb) [file 13287_2016_419_MOESM3_ESM.pdf]

Supplementary Table S1. Gene Expression

Gene	MSC (n = 8 High-GAG replicates)					ACP (n = 8 High-GAG replicates)				
	20% O_2		2% O_2			20% O_2		2% O_2		
	Gene Expression	SD	Gene Expression	SD	*p-value	Gene Expression	SD	Gene Expression	SD	*p-value
COL2A1	1.07 E -03	8.39 E -04	2.34 E-03	2.15 E-03	**0.012**	4.00 E -03	4.17 E -03	1.02 E -02	1.09 E -02	0.100
COL9A1	7.43 E-04	1.23 E-03	2.03 E-03	3.40 E-03	**0.011**	1.05 E -04	2.95 E -04	5.33 E -04	6.76 E -04	0.304
COL11A2	9.44 E-04	1.20 E-03	2.81 E-03	4.47 E-03	**0.008**	3.94 E -04	9.95 E -04	1.31 E -03	1.69 E -04	**0.004**
COL6A1	1.34 E-03	1.76 E-03	1.73 E-03	1.76 E-03	**0.005**	2.45 E -04	2.78 E -04	3.23 E -04	3.61 E -04	**0.036**
ACAN	8.76 E-04	9.12 E-04	1.82 E-03	1.89 E-03	**0.010**	3.99 E -04	5.46 E -04	1.28 E -03	1.44 E -03	**<0.001**
PRG4	1.40 E-05	2.50 E-05	1.45 E-04	2.44 E-04	**<0.001**	1.10 E -03	1.97 E -04	1.28 E -02	2.84 E -02	**0.054**
L-SOX5	5.36 E-05	7.36 E-05	1.99 E-04	3.09 E-04	**0.001**	6.13 E -04	7.31 E -04	1.03 E -03	1.50 E -03	0.236
SOX6	1.22 E-04	1.96 E-04	1.96 E-04	3.01 E-04	0.065	4.24 E -04	1.50 E-04	8.47 E -04	3.00 E -04	**0.022**
SOX9	1.04 E-04	1.44 E-04	2.65 E-04	4.43 E-04	**0.005**	4.05 E -05	3.57 E-05	8.70 E-05	9.35 E -05	**0.03**
LOX	7.37 E-03	1.20 E-02	6.96 E-03	1.25 E-02	0.080	3.21 E -03	4.48 E-03	7.55 E-03	1.20 E -02	**<0.001**
COL1A1	3.33 E-02	6.70 E-02	2.46 E-02	5.25 E-02	**0.002**	6.12 E-02	8.27 E-02	6.66 E-02	1.06 E-01	0.549
COL10A1	1.77 E-03	1.50 E-03	9.46 E-04	5.47 E-04	**0.035**	1.04 E-02	1.68 E-02	3.68 E-04	6.04 E-04	**0.001**
MMP13	8.26 E-05	9.77 E-05	2.20 E-05	3.80 E-05	**0.011**	1.55 E-03	2.08 E-03	1.08 E-04	1.53 E-04	**0.001**

Gene Expression relative to *18S* housekeeping gene. *Significance determined by paired t-test of gene expression in hyperoxia (20% O_2) and physioxia (2% O_2)
